# Physicochemical analysis of rotavirus segment 11 supports a ‘modified panhandle’ structure and not the predicted alternative tRNA-like structure (TRLS)

**DOI:** 10.1007/s00705-013-1802-8

**Published:** 2013-08-13

**Authors:** Subhajit Biswas, Wilson Li, Emily Manktelow, Jonathan Lever, Laura E. Easton, Peter J. Lukavsky, Ulrich Desselberger, Andrew M. Lever

**Affiliations:** 1 Department of Medicine, University of Cambridge, Level 5, Addenbrooke’s Hospital, Hills Road, Cambridge, CB2 0QQ UK; 2 Structural Studies Division, Medical Research Council (MRC) Laboratory of Molecular Biology, Cambridge, CB2 0QH UK; 3 Present Address: School of Life Sciences, University of Lincoln, Witham House, Brayford Pool, Lincoln, LN6 7TS UK

## Abstract

Rotaviruses are a major cause of acute gastroenteritis, which is often fatal in infants. The viral genome consists of 11 double-stranded RNA segments, but little is known about their *cis*-acting sequences and structural elements. Covariation studies and phylogenetic analysis exploring the potential structure of RNA11 of rotaviruses suggested that, besides the previously predicted “modified panhandle” structure, the 5’ and 3’ termini of one of the isoforms of the bovine rotavirus UKtc strain may interact to form a tRNA-like structure (TRLS). Such TRLSs have been identified in RNAs of plant viruses, where they are important for enhancing replication and packaging. However, using tRNA mimicry assays (*in vitro* aminoacylation and 3’- adenylation), we found no biochemical evidence for tRNA-like functions of RNA11. Capping, synthetic 3’ adenylation and manipulation of divalent cation concentrations did not change this finding. NMR studies on a 5’- and 3’-deletion construct of RNA11 containing the putative intra-strand complementary sequences supported a predominant panhandle structure and did not conform to a cloverleaf fold despite the strong evidence for a predicted structure in this conserved region of the viral RNA. Additional viral or cellular factors may be needed to stabilise it into a form with tRNA-like properties.

## Introduction

Rotaviruses (RVs) are a major cause of acute gastroenteritis in infants and young children worldwide and are responsible for considerable mortality, mainly in developing countries. Rotaviruses form a genus of the family *Reoviridae* and are icosahedral triple-layered particles (TLPs). They contain a genome of 11 segments of double-stranded RNA encoding six structural viral proteins (VP1-VP4, VP6 and VP7) and six non-structural viral proteins (NSP1-NSP6). After infection, enzymatic digestion of the outer particle layer in the cytoplasm produces double-layered particles (DLPs) that are transcriptionally active and extrude 11 ssRNA segments of positive polarity. These RNA molecules are either translated into viral proteins or function as templates for RNA replication, producing the double-stranded genome in progeny virions. Early viral morphogenesis and RNA replication occur in cytoplasmic inclusion bodies called viroplasms [1].

The molecular mechanisms by which rotaviruses select and package 11 distinct ssRNA segments in equimolar amounts into every virion from the RNA pool in each viroplasm are poorly understood. Similarly, regulation of the balance between virus RNA replication (for packaging) and viral protein translation remains obscure, although *cis*-acting signals in the RNA are likely to control this [1]. Rotavirus RNA11 (667 nt) codes for two non-structural proteins, NSP5 and NSP6, and the coding sequences for these proteins have overlapping reading frames. Previously, prediction of the consensus secondary structure of positive-sense RNA11 suggested the presence of a “modified panhandle” structure involving long-range interactions (LRIs) formed between sequences close to the 5’ and 3’ termini of the RNA [2]. However, two observations point strongly towards an alternative model for the terminal structure of this molecule. Firstly, the conserved LRIs between sequences at nt 18-42 and nt 617-638 are unusually frequently interrupted by bulges and small loops compared to other rotaviral RNA segments. Secondly, while the sequence of the 5’ strand within this region, much of which is coding, is highly conserved, the sequence of the 3’ strand (which is entirely non-coding and within the 3’-UTR) has unusually low sequence conservation. Most strikingly, there are two sites within the 3’ strand that demonstrate a very high level of co-variation, consistent with base pairing in the 3’ region, which would trigger an equivalent pairing in the 5’- strand to create a cloverleaf structure resembling a tRNA fold. Notably, biochemical structure mapping studies of this region are also consistent with two alternative structures [2].

Transfer-RNA-like structures (TRLSs) have so far only been identified in plant virus RNA, such as in turnip yellow mosaic virus (TYMV) and brome mosaic virus (BMV), where they are important for enhancing replication, packaging and other functions [3, 4]. Transfer RNAs are ancient molecules present in all domains of life. Typically tRNAs adopt a “cloverleaf” secondary or “L”-shaped 3D structure, comprising an acceptor arm, D-arm, T-arm and an anticodon arm, and contain several modified ribonucleotides such as dihydrouridine and pseudouridine [5]. The single-stranded CCA tail at the 3’ end of the acceptor arm is important for the recognition of the tRNAs by a specific aminoacyl tRNA synthetase, which precedes attachment to the cognate amino acid. Besides translating the genetic code into protein, tRNAs perform many other functions such as RNA folding, structure stabilization, hydrolysis and lipid remodelling [5].

In eukaryotes and archaea, unlike prokaryotes, the 3’ CCA sequence is not encoded by the tRNA gene. The CCA-adding nucleotidyl transferase (CCA-NTase) synthesizes and regenerates the 3’ CCA sequence of tRNA by adding three consecutive nucleotides in the order C, C, and A in a primer-dependent but template-independent fashion. This is accomplished by a process of “collaborative templating” in which the enzyme and the bound tRNA substrate jointly specify the identity of the next nucleotide to be added [6]. The ability of CCA-adding enzymes to recognize all cytoplasmic tRNAs regardless of amino acid acceptor specificity suggests that recognition involves structural features common to most tRNAs. The enzyme is essential in eukaryotes and archaea, in which the 3’ trailer sequences are removed from tRNA precursors by nucleases that stop at the discriminator base (position 73), leaving the tRNA acceptor stem intact as a substrate for the CCA-adding enzyme [7]. TYMV RNAs are known to have 3’ TRLS and can be readily adenylated by host or wheat germ CCA-NTase [8]. The majority of plant virus TRLSs can also be aminoacylated by aminoacyl tRNA synthetases in the presence of their cognate amino acids [3]. Recognition of the appropriate tRNA by the synthetases is mediated by the tRNA anticodon and the acceptor stem.

Given the striking structural and phylogenetic evidence of such a TRLS identified for the first time in an animal virus, we sought to evaluate how similar it was in its properties to previously identified TRLSs of plant viruses. We attempted *in vitro* aminoacylation and CCA nucleotidylation of the intact segment and of an internally deleted version, which was easier to manipulate and potentially more sensitive as a substrate. NMR studies were also conducted on a deletion construct of RNA11, comprising 5’- and 3’-terminal sequences (including the sequences that can potentially form intra-strand base-pairing). This construct was joined by a stable tetraloop on one end of the helix and clamped by three additional G-C pairs at the other end. Despite the structural evidence of a TRLS in this RNA, we could not demonstrate typical biochemical properties of tRNA in RNA11.

## Materials and methods

### Alignment of rotavirus RNA11 sequences and structure analysis

**Fig. 1:**
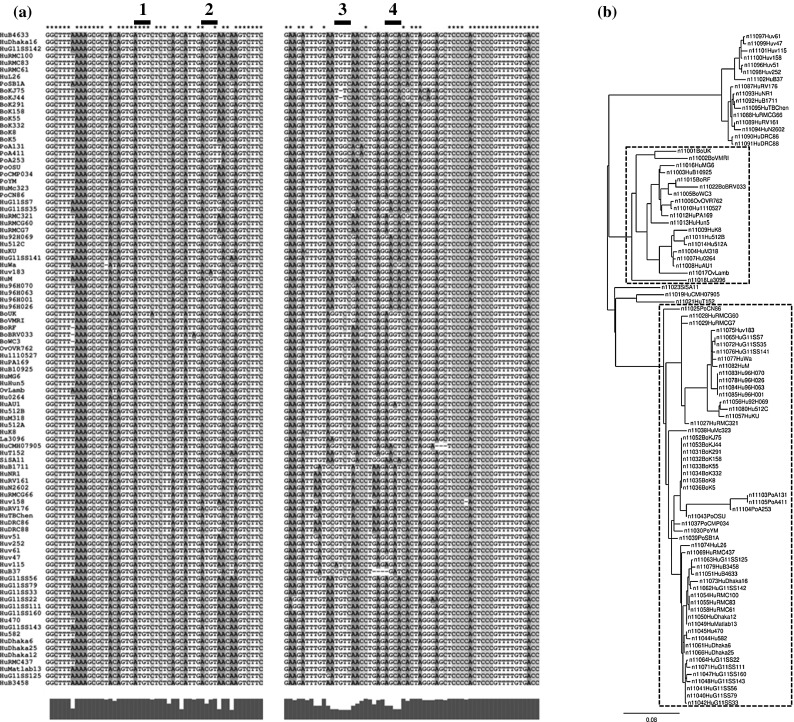
Sequence alignment of the 5’- and 3’-terminal regions of RNA11. **a** The variation found at each nucleotide is depicted graphically below the alignment. Nucleotides that are fully conserved in all strains are marked with an asterisk. The 5’ stem-loop (SL) is formed between sites 1 and 2 in the highly conserved 5’-terminal region. The 3’-SL is formed between sites 3 and 4 in the 3’-terminal region with low sequence conservation but exhibits co-variation. A 3’-SL with 3- or 4-bp stem-loops can be formed in >95 % of isolates. **b** Phylogenetic tree of RNA11. The two large groups of sequences analysed using RNAalifold are marked by the *dotted line*. The *scale bar* indicates nucleotide substitutions per site

The accession numbers of all 94 group A RV RNA11 full-length sequences retrieved from GenBank and used for alignment by ClustalX2 (Fig. 1a) have been published elsewhere [2] . A maximum-likelihood phylogenetic tree (Fig. 1b) was generated from the alignment using a web server (http://www.phylogeny.fr, accessed 31-05-2013). Sequences from two phylogenetic groups were used for the RNAalifold analysis (Fig. 1). RNAalifold analyses were done using the new version of RNAalifold without the RIBOSUM scoring option [9]. The penalty for non-compatible sequences was set at 0.5, and the weight of covariance term was set to 1 unless mentioned otherwise. For analysis of both 5’- and 3’-terminal regions, alignments of the two sequences were linked with 5 nt forced to be unpaired by folding constraints. Minimum-free-energy (MFE) structures were predicted using RNAfold [10].

### RNA transcription and purification

The cDNA of boRV UKtc strain gene 11 has been cloned previously into the multi-cloning site of the TA cloning vector pCR2.1 from Invitrogen (plasmid kindly provided by Malcolm McCrae, University of Warwick, UK). PCR amplification from this plasmid generated positive-sense cDNA with a T7 promoter immediately preceding the 5’ terminus of gene 11. The following primers were used: 5’TAATACGACTCACTATAGGCTTTTAAAGCGCTACAGTG3’ ((+) sense; T7 promoter sequence underlined) and 5’GGTCACAAAACGGGAGTGGGG3’ ((-) sense) [2].

Rotavirus RNA11 of positive polarity with authentic 5’ and 3’ termini was subsequently obtained from the above PCR-amplified cDNA by *in vitro* transcription using T7 polymerase and an Ambion MEGAscript T7 transcription kit, following the manufacturer’s instructions. Per transcription reaction, 0.5-1.0 μg cDNA was used, and the reaction was carried out for up to 3.5 h at 37 °C. Subsequently, 1 μl of DNase Turbo (1 U/μl, Ambion) was added directly to the transcription reaction, which was incubated for a further 15 minutes at 37°C. RNA was then purified by phenol/chloroform extraction, followed by precipitation with ethanol in the presence of 0.5M NH_4_OAc. The size and integrity of the RNA were confirmed by polyacrylamide gel electrophoresis, and the RNA concentration was determined using a NanoDrop^®^ ND-1000 UV-Vis spectrophotometer.

To transcribe RNA11 with an additional 3’ A, the above (-) sense primer for PCR was redesigned with a thymidine added at its 5’ end. Subsequent *in vitro* transcription using this PCR product resulted in generation of RV RNA11 with a 3’ A. Adding a cap analogue at the 5’end of the 3’-adenylated RNA11 was accomplished by *in vitro* transcription as described above using an Ambion mMESSAGE mMACHINE capped transcription kit, following the manufacturer’s instructions.

The cDNA of HIV-2 ROD was cloned previously into pSVR∆NB [11]. PCR amplification from this plasmid generated positive-sense cDNA (751 bp) with a T7 promoter immediately preceding the 5’ terminus of the gene. The primers used were 5’TAATACGACTCACTATAGGTCGCTCTGCGGAGAGGCTGG3’ ((+) sense; T7 promoter sequence underlined) and 5’GGTACCATTGGATCTAAAACTG3’ ((-) sense). HIV-2 ROD RNA (751 bases) was obtained by *in vitro* transcription and purification as described above and used as a negative control in the tRNA mimicry assays.

### RNase T1 probing of 5’-end-labelled rotavirus RNA11

Purified RNA11 molecules were radiolabelled at the 5’ terminus using [γ-^32^P] ATP (Perkin Elmer) and a KinaseMax 5’- End Labelling Kit (Ambion) following manufacturer’s instructions. Radiolabelled RNA11 molecules were purified using RNeasy spin columns (QIAGEN). To ensure structural uniformity, RNA was heated to 80°C for 10 minutes and allowed to cool to room temperature before partial cleavage by RNase T1 (Ambion), which cleaves unpaired guanine residues. Reactions were incubated at room temperature for 10 minutes in 10-μl reactions containing 1 μg carrier yeast tRNA (Ambion AM7119). The reactions were stopped by the addition of inactivation/precipitation buffer (supplied with RNase T1 by Ambion). Each reaction contained 2 μg of RNA11. Samples were analysed on 12 % polyacrylamide 7 M urea gels. The sequence ladder was generated from RV RNA11 by alkaline hydrolysis.

### 3’-Adenylation assay

The CCA-NTase activity of HeLa S100 fraction (Enzo Life Sciences) was tested by the incorporation of [α-^32^P] ATP into a yeast tRNA mixture or a crude wheat germ tRNA preparation (Type V, Sigma-Aldrich R7876). Approximately 4 % of the tRNA molecules in these crude tRNA preparations have incomplete 3’ CCA termini and therefore act as substrates for 3’ adenylation by CCA-NTase [12]. Standard assays were performed as described previously [12] in 10-μl reactions containing 100 mM glycine/NaOH, pH 9.0, 10 mM MgCl_2_, 1 mM DTT, 2-4 μg tRNA, 100 μM of each rNTP, 0.033 μM [α-^32^P] ATP (3000 Ci/mol, 10 mCi/ml), and HeLa S100 fraction (1.0 μg). The latter is used as a source of tRNA-reactive enzymes [13].

The control tRNAs and test RNAs were first heated to 85 °C and then cooled at room temperature for 15 min before being used in the assay. Incubation was at 37 °C for 40 min. The RNA from individual reaction mixes were purified using the phenol-chloroform extraction method and analyzed by denaturing gel electrophoresis (7 M urea, 12% polyacrylamide). The sizes of the resolved RNA bands on the gel were determined by comparison to an RNA ladder, comprising single-stranded RNA transcripts (RiboRuler Low Range, Fermentas). Gels were first stained with ethidium bromide (1 μg/ml) to visualize the RNA bands by UV transillumination and then subjected to autoradiography to detect 3’ radiolabelling of RNAs.

### Aminoacylation assay

The aminoacylation assay followed published protocols [14, 15]. Reactions were performed at 37 °C for 1 h in a reaction buffer (pH 7.6) consisting of 0.1 M Tris HCl containing 5, 10 or 20 mM MgCl_2_, 50 mM KCl, 0.5 mM EDTA and 2.5 mM ATP. The RNAs were first denatured at 80°C and cooled at room temperature as described above. The 50-μl final reaction mixture contained 5 or 10 μg control tRNA or different concentrations of test RNA, 1-3 μl (0.1 μCi/μl) of a ^14^C-labelled amino acid mixture of 15 L-amino acids (Perkin-Elmer, NEC-445) and HeLa S100 fraction (approximately 10-15 μg/reaction). Higher quantities of HeLa S100 fraction were applied in tests with larger amounts of tRNA used. This was done to maximize the catalytic activity of aminoacyl tRNA synthetases and increase the margin of fold-binding of amino acid, if any, between the presence and absence of the synthetases.

Following incubation, 20 μl aliquots were spotted on 25-mm GF-C filter discs (Whatmann) and left at room temperature until they had dried. A second 20-μl aliquot/reaction was pipetted into 200 μl chilled 10% trichloroacetic acid (TCA) and left on ice for 2 h to precipitate the RNA. The unincorporated ^14^C-amino acids were removed by filtering through GF-C filters under vacuum, using four washes with 500 μl of 5 % TCA, followed by 500 μl of 70 % ethanol, and the filter discs were then dried. The two sets of filter papers were then individually immersed in liquid scintillation cocktail (Optiphase Hisafe 2), and the amounts of radiolabelled amino acids bound were measured in a liquid scintillation analyzer (Tri-Carb 2100TR, Packard). The counts (dpm) of the second set of washed filters (resulting from the “bound” amino acids) were expressed as a percentage of the total count from the corresponding unwashed filter papers.

Differences between sets of data (e.g., percent bindings of yeast tRNA with and without HeLa cell extract) were compared using Student’s t-test (two-tailed), and the variance of the data over several experiments was measured by the F test. A value of *P*<0.05 was considered to be statistically significant.

### Generation of a boRV RNA11 5’-3’- deletion construct, joined by a UUCG loop

An RNA deletion construct (117 bases) of RV RNA11 was transcribed comprising the first 52 nt at the 5’ end and the last 61 nt at the 3’ end, joined by an artificial loop-forming sequence 5’-…UUCG…-3’. For this, two overlapping DNA templates were synthesized [(forward S1: 5’AGCTAGAAGAATTCATAT**TAATACGACTCACTATAG**GCTTTTAAAGCGCTAAAGTGATGTATCTCAGTATTGACGTGacgagtcttcttcggaagatttgtaggtctga3’) and (reverse S2: 5’TTTTTTTTAAGCTTTTTTCTGCAGGTCACAAAACGGGAGTGGGGAGCTCCCTAGTGACCTCTCAGGtcagacctacaaatcttccgaagaagactcgt3’)] (Sigma-Aldrich).

The forward template (S1, 109 nt) contained an Eco RI restriction site in a 5’-3’ direction (underlined in the S1 sequence) and a T7 promoter sequence downstream (shown in bold in the S1 sequence), separated by four spacer nucleotides. The first 74 nt of the target 117-nt construct immediately followed the T7 promoter sequence. The reverse template (S2, 98 nt) was designed such that reverse complementarities (S2-reverse-complementary: 5’acgagtcttcttcggaagatttgtaggtctgaCCTGAGAGGTCACTAGGGAGCTCCCCACTCCCGTTTTGTGACCTGCAGAAAA**AAGCTT**AAAAAAAA3’), in a 5’-3’ direction, would produce a 32-nt overlapping sequence with the 3’ end of S1 (shown in lower case in S1, S2 and S2 reverse-complementary), followed by the last 43 nt of the target construct ending in -CC. The last ‘C’ was the starting ‘C’ of a Pst I restriction site (underlined in the S2 reverse-complementary sequence). This is followed by the Hind III restriction site (shown in bold in the S2-reverse-complementary sequence), spaced between two stretches of spacer sequences.

The S1 and S2 DNA templates were used in a “hot-start PCR” reaction, using *Pfu* DNA polymerase (Promega) with forward (SB1: 5’AGCTAGAAGAATTCATATTAATA 3’) and reverse (SB2, 5’TTTTTTTTAAGCTTTTTTCTGC3’) primers to produce a PCR product for cloning into the pUC19 vector (Invitrogen). The cycle conditions were as follows: initial melting at 99 °C for 1 min and one cycle of 99 °C for 30 s, 55 °C for 30 s and 72 °C for 10 min. This was followed by 30 cycles of 94 °C for 1 min, 55 °C for 30 s and 72°C for 2 min. The final extension cycle comprised incubation at 72 °C for 10 min. The resulting PCR product (175 bp) was cloned into pUC19 using restriction sites Eco R1 and Hind III. The pUC19 construct was then expanded by transforming and culturing *E.coli* DH-5α cells, followed by plasmid purification (QIAGEN Plasmid Maxi Kit, following manufacturer’s instructions). The plasmid was then linearized by Pst I digestion, followed by removal of the 3’ overhang using T4 DNA polymerase (Promega) to obtain linear DNA terminating in the authentic rotavirus 3’ sequence of 5’…ACC-3’. This linearized plasmid was used as a template for obtaining the target 117-base RNA construct by *in vitro* transcription (T7-MEGAscript Kit, Ambion, following manufacturer’s instructions).

### RNA NMR sample preparation and NMR spectroscopy

A DNA template containing the T7 promoter, the desired RNA sequence, a two-nucleotide linker and a BbsI restriction site (to allow for effective run-off transcription) flanked by HindIII and EcoRI restriction sites (to allow subcloning into the pUC18 multiple cloning site) was generated by PCR amplification using the following overlapping primers: 5’GAGCAAGCTTAATACGACTCACTATAGGCAGCAGT**GCAGTTATGACTC TCTGTAGTGACTTC**3’ (T7 promoter sequence underlined) and 5’GCTCTCTA GAGAAGACAAGGCAACATCCAGACTGGACTCTCCAGTGACC**GAAGTCACTACAGAGAGTCATAACTGC**3’ (overlapping sequences shown in bold) [16]. The template was then subcloned into the multiple cloning site of the vector pUC18 and introduced by transformation into *E.coli* DH5α cells, which were used to produce milligram quantities of the recombinant plasmid. The plasmid was purified using a QIAfilter Plasmid Mega Kit (QIAGEN) and then linearised with BbsI before performing *in vitro* transcription using T7 RNA polymerase. The RNA transcripts were purified using weak anion-exchange FPLC [17], and the purified RNA fractions were then pooled, concentrated and equilibrated in 10 mM sodium phosphate buffer, pH 6.0 using 15 ml Centriprep centrifugal concentrators with a 10,000-Da molecular weight cutoff. NMR samples were prepared in Shigemi NMR tubes (280 μl containing 5 % D_2_O (v/v) and 0.25 mM d_12_-EDTA) at an RNA concentration of 0.5 mM. NMR data were acquired at 25°C on a Bruker Avance 800 spectrometer equipped with a 5-mm cryo-probe. Base pairing schemes were established from NOE patterns in 2D NOESY spectra recorded in 95 % H_2_O/5 % D_2_O (v/v) [18].

## Results

### boRV RNA11 can form a tRNA-like cloverleaf structure

Phylogenetic analysis of the sequences at the 5’ and 3’ termini of RNA11 of 94 group A RV strains identified two regions in the 3’ arm that showed both much higher levels of nucleotide variation than the surrounding sequences and also strong evidence of co-variation (Fig. 1). Previously, using single-sequence methods such as MPGAfold [19] or comparative methods such as ConStruct [20], RV RNA11 has been suggested to form a “modified panhandle” structure with extended long-range interactions (LRIs) between the 5’- and 3’-terminal regions [2]. However, most of these programmes do not allow the prediction of tertiary structures such as pseudoknots or kissing-loop conformations. It is also difficult to predict alternative conformations that are only stabilised under certain *in vivo* conditions such as protein binding and physiological concentrations of divalent cations.

**Fig. 2:**
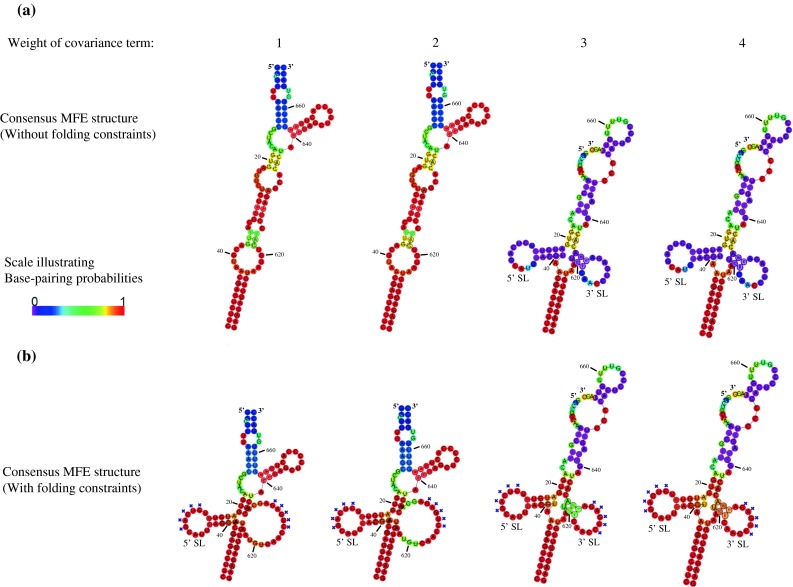
RNAalifold suggests an alternative cloverleaf model for rotavirus RNA11. **a** Alignments of RNA11 sequences from the two largest phylogenetic groups (Fig. 1) were analysed using RNAalifold with weight of covariance term from 1 to 4. **b** The same analysis as in panel a except that the two potential tertiary interaction sites (marked by *blue crosses*) are forced to be single-stranded. Structures were illustrated using the consensus sequence. Nucleotide positions correspond to that of the bovine rotavirus UKtc strain. *Colours* represent base-pairing probabilities illustrated by the *colour scale*

**Fig. 3:**
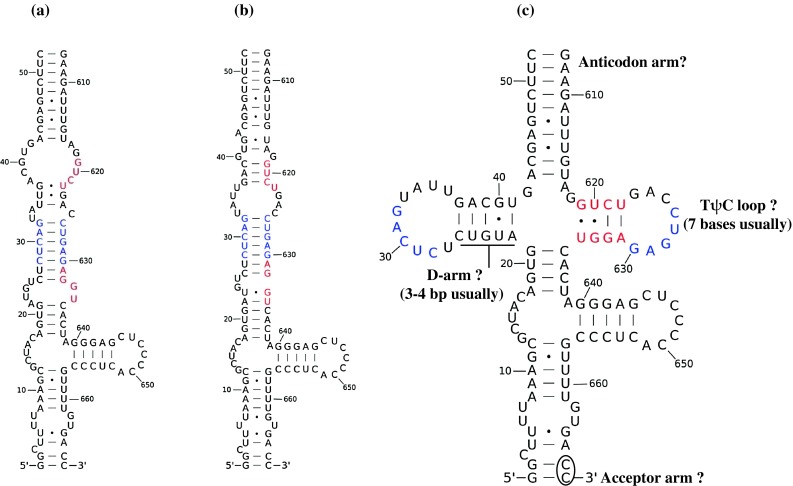
Structures predicted for the termini of RNA11 of the bovine rotavirus UKtc strain. **a** The panhandle model for RNA11, illustrated by the consensus secondary structure predicted by RNAalifold without using folding constraints. **b** Terminal MFE structure of RNA11 of the bovine rotavirus UKtc strain predicted by RNAfold. **c** The cloverleaf model for RNA11, illustrated by the consensus secondary structure predicted by RNAalifold using base-pairs in 5’-SL and 3’-SL as folding constraints. Nucleotides labelled in *blue* in panel b have the potential to form tertiary interactions. Nucleotides labelled in *red* are those that show high co-variation and when paired together result in the formation of the cloverleaf structure

When the alignment of sequences from the two largest phylogenetic groups of RNA11 (Fig. 1) were analysed using RNAalifold [9] with increasing weight of the covariance term, the consensus structure prediction shifted from the panhandle model to a cloverleaf model involving a 4-helix junction. This shift occurred when the weight of covariance term was greater than 2 (Fig. 2a). The cloverleaf model involves a 5’ stem-loop (5’-SL, nt 22-41) in the highly conserved 5’-terminal region, and a 3’ stem-loop (3’-SL, nt 619-634) in the less conserved 3’-terminal region. The 3’-SL involves a helix held by covariation between two small regions of high sequence variation (Fig. 2a). Evaluation of the sequence alignment led to the finding that 89 out of 94 RNA11 sequences can form a short helix with at least three base pairs (Fig. 1). Because the loop sequences of 5’-SL and 3’-SL are base-paired in the panhandle model but not in the cloverleaf model, the 4-helix junction could be stabilised by kissing-loop interactions. However, the RNAalifold algorithm is unable to solve tertiary interactions. To mimic this interaction, the RNAalifold analysis was repeated, but with the potential tertiary interaction sites forced to be unpaired. 5’- SL was then predicted with a weight of covariance term as low as 1, while the 3’-SL was predicted with much higher base-pairing probabilities (Fig. 2b). At a weight of covariance term of 1, the panhandle model was predicted by RNAalifold (Fig. 3a). This resembles the minimum-free-energy (MFE) structure of RNA11 of the bovine rotavirus UKtc strain (Fig. 3b). The cloverleaf model (Fig. 3c) predicted by RNAalifold using base pairs from both the 5’-SL and the 3’-SL as folding constraints only differs from the panhandle model at nt 22-41 and nt 619-634.

**Fig. 4:**
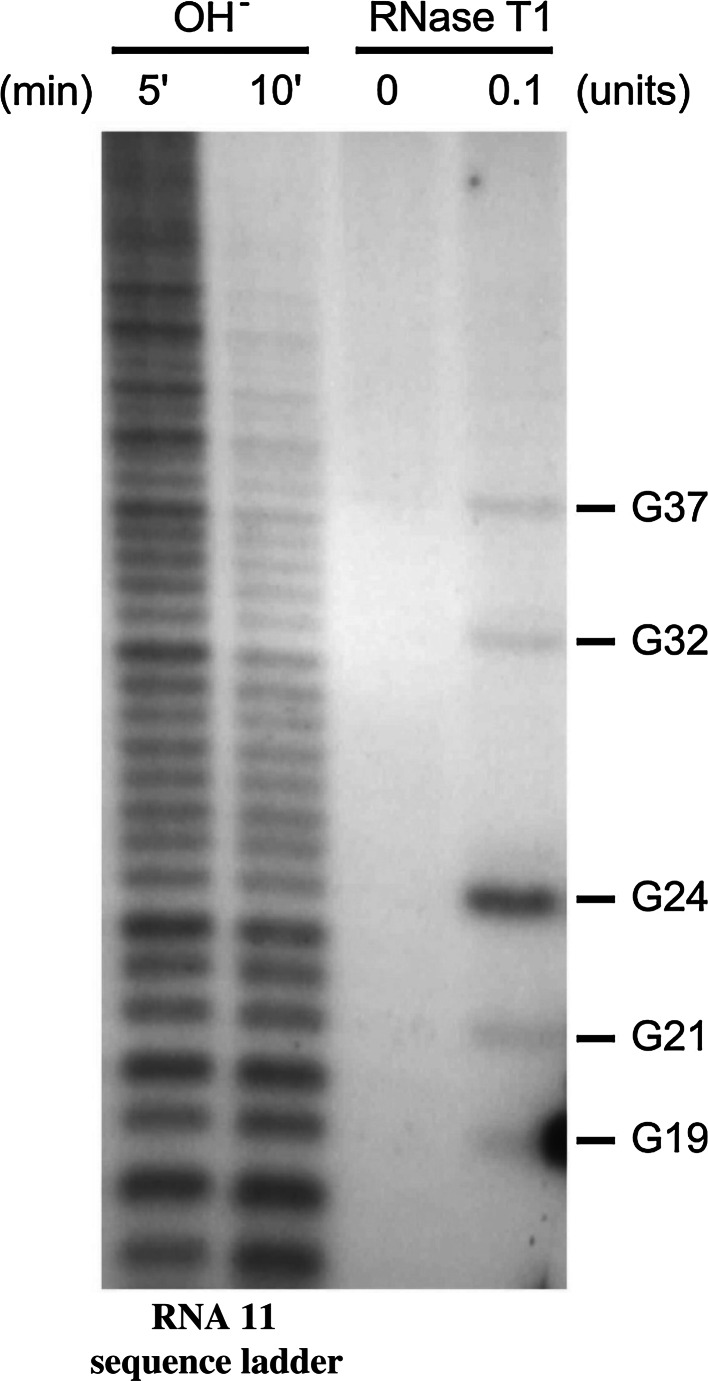
RNase T1 cleavage results of single-stranded guanines in the 5’-terminal sequence of rotavirus RNA11. Single-stranded rotavirus RNA11 was labelled at the 5’ terminus using [γ-^32^P]ATP, subjected to partial digestion by RNase T1, and resolved on a 12 % polyacrylamide 7 M urea gel. The *dark bands* in the last column on the right show the positions of the single-stranded guanines cleaved by RNase T1, compared to the third column from the left (no-enzyme control). The first and second columns from the left represent an RNA11 sequence ladder generated from the same RNA by alkaline hydrolysis for 5 and 10 min, respectively

In the panhandle model, G32 was predicted to form a G-C pair in a stable helix (Fig. 3a and b). However, in the 5’-end labelling experiment, G32 showed weak sensitivity to RNase T1 digestion (Fig. 4). Therefore, G32 may exist as a single-stranded nt, as would occur in the loop region shown in the cloverleaf model (Fig. 3c). In the panhandle model, G24 is present as a single-stranded nt, while in the cloverleaf model it is at the centre of a stable helix. The stronger RNase T1 cleavage at G24 compared to that at G32 suggests that although many of the RNA molecules adopt the panhandle structure *in vitro*, a small proportion exists in an alternative tRNA-like conformation, perhaps in equilibrium with the predominant panhandle structure. In the cloverleaf/tRNA model, the shorter panhandle could mimic the anticodon arm of the tRNA, with the 5’ and 3’ ends of RNA11 being found in the acceptor arm. The D-arm stem equivalent (the base-paired region in the 5’ arm of the cloverleaf) is 5 bp long (normally 3-4 bp in tRNAs), while the T-loop equivalent is 8 bases long (usually 7 bases in tRNAs). Furthermore, the 5’…C_28_UCAG_32_…3’ sequence in the putative D-loop can potentially base-pair with the 3’…G_630_AGUC_626_…5’ sequence in the putative T-loop (Fig. 3c) resulting in the D- and T-loop kissing interaction as is almost invariant in tRNA.

The cloverleaf MFE structure of RNA 11 consensus sequence predicted with or without folding constraint (Fig. 2) terminates in an unpaired 3’- CC, similar to the single stranded 3’- CCA observed in the case of tRNAs of eukaryotes and archaea [6, 7]. This is further supported by the observation that for virus replication to take place, the 3’ end of each rotavirus mRNA has to be single-stranded [19] so as to engage with the VP1 RNA-dependent RNA polymerase (RdRp) [21–24]. Like RV mRNAs, TYMV or BMV TRLSs also possess a single-stranded 3’-CC [25]. Following 3’-adenylation by CCA-NTase, the TRLSs of these plant virus RNAs serve as substrates for valylation, a pre-requisite for TYMV infectivity [4] or optimum BMV RNA replication [26]. Therefore, we carried out *in vitro* assays to search for similar TRLS functions in RV RNA11.

### boRV RNA11 was not 3’-adenylated by CCA-NTase activity of the HeLa S100 fraction

**Fig. 5:**
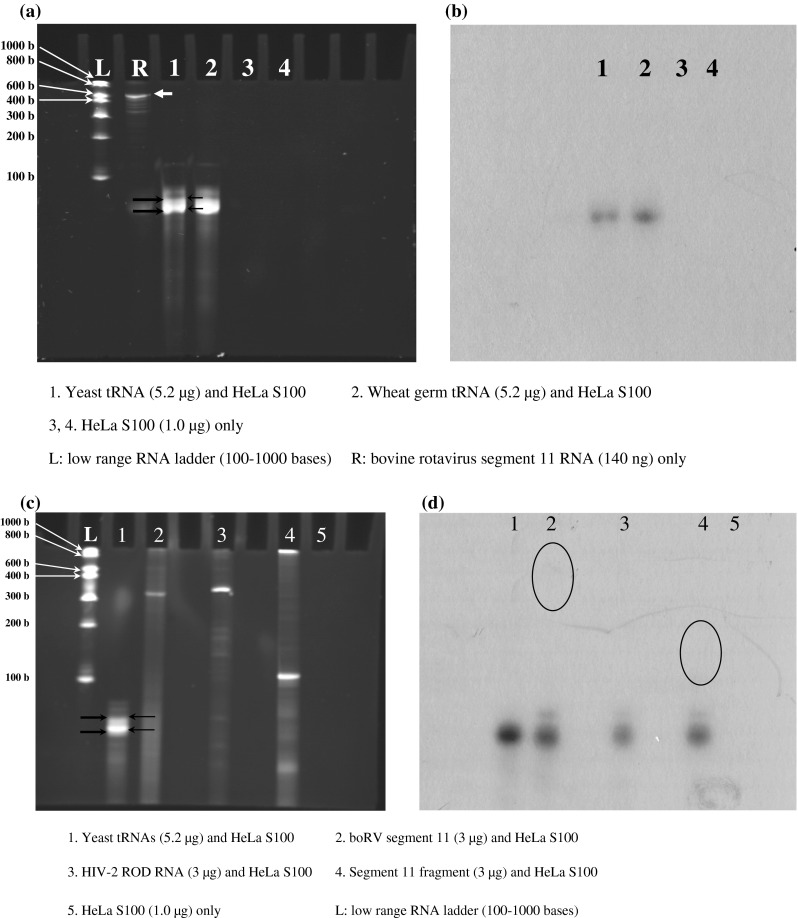
Testing adenylation with eukaryotic tRNAs, RNA11 or its 5’-3’construct at their 3’ end by CCA-NTase activity present in HeLa S100 fraction **a** PAGE (12 % polyacrylamide, 7 M urea) showing ethidium-bromide-stained bands of control tRNAs and “no RNA only HeLa S100” controls following 3’ adenylation and phenol/chloroform purification of RNA as well as the RNA11 band (no HeLa S100 added). **b** Autoradiograph of the gel shown in panel a, demonstrating RNAs that have been 3’-denylated with [α-^32^P] ATP. **c** PAGE (12 % polyacrylamide, 7 M urea) showing ethidium-bromide-stained bands of control and test RNAs following 3’adenylation reactions and phenol/chloroform purification of the RNAs from reaction mixtures. **d** Autoradiography of the same gel in panel c, showing RNAs that have been 3’-adenylated with [^α−32^ P] ATP. The estimated concentration of test RNAs is 3-4 μg. The *thick black arrows* on the gel indicate the position and width of the bipartite tRNA band visible by ethidium bromide staining, while the thin *black arrows* indicate the same for the corresponding band on the autoradiograph. The position of migration of the RNA11 band (without HelaS100 being added) is indicated by the *thick white arrow*. The circles encompass the areas where radiolabelling was expected to be observed for the RNA11 or its 5’-3’ construct, had they been 3’-adenylated. Following adenylation reactions using HeLaS100 fraction and subsequent RNA extraction, both RV RNA11 and HIV-2 ROD RNAs were found to migrate faster (observations from repeated experiments), as is evident from the position of the ethidium bromide-stained bands (Fig. 5a and c). The reason for this observed shift in migration is not clear

*In vitro* 3’-adenylation experiments were successful with eukaryotic tRNAs from yeast or wheat germ, using HeLa S100 fraction as a source of the CCA-NTase (Fig. 5a and b). The poly (A) polymerase is the only other known RNA polymerase that adds 3’ A in a primer-dependent but template-independent manner. But unlike CCA-NTase, poly (A) polymerase is promiscuous and synthesizes products that are heterogeneous in length [12]. Unlike poly (A) polymerase, the CCA-NTase displays remarkable specificity, uniquely recognizing tRNA as substrate and adding a precisely defined 3’-terminal CCA sequence. In all our experiments, the incorporated label in denaturing polyacrylamide gels coincided with the bipartite tRNA detected by ethidium bromide staining. The data shown in Fig. 5a and b conclusively proves that the 3’-labelling of the tRNAs is mediated by CCA-NTase activity and not by poly (A) polymerase.

Attempts to incorporate radiolabelled [α-^32^P] ATP at the …CC-3’- end of RNA11 proved unsuccessful. No radiolabelling could be detected in the radiographs in the expected area (evident from visible bands by ethidium bromide staining in Fig. 5c) even after prolonged exposure (up to 20 days). This observation was further strengthened by the fact that only a small proportion (~4 %, i.e., estimated 200 ng) of the yeast tRNAs (5.0 μg) used as a positive control were authentic substrates with 3’- CC for adenylation [12], while 100 % of RNA11 (estimated 350 ng to 3.0 μg) should have …CC-3’ and therefore be available for 3’-adenylation. HIV-2 ROD RNA, used as a non-specific RNA control, was not 3’-adenylated, either, as expected (Fig. 5d).

### boRV RNA11 was not aminoacylated

A eukaryotic yeast tRNA mixture (Ambion), used as a positive control, showed effective aminoacylation with ^14^C-labelled amino acid (s) in the presence of HeLa S100 (in comparison to reactions in which the cell extract was absent). The mean proportion of bound tRNA (i.e., aminoacylated) expressed as a percentage of both bound and unbound tRNAs was 12.2 ± 1.9 % (mean ± standard deviation, calculated from 5 independent experiments). Compared to this, the nonspecific binding of yeast tRNAs and ^14^C-amino acids in the absence of any aminoacylating enzyme was 2.2 ± 0.8 % (from 5 experiments). The addition of HeLa S100 increased the binding by more than fivefold compared to when the cell extract was not added to the tRNA and amino acid mixtures, and this difference was statistically significant (*P*<0.001). The radiolabelled amino acid mixture in the absence of any RNA or enzyme also produced similar nonspecific binding, i.e., 2.3 ± 0.8 % with the filter paper following washing (data from 4 experiments). Incubation of the HeLaS100 fraction only in the presence of radiolabelled amino acids produced nonspecific binding of 1.0 ± 0.4 % for 2 μl and 0.7 ± 0.04 % for 3 μl of the fraction.

The binding of boRV RNA11 with radiolabelled amino acids ranged from <1 to 2.2-fold compared to the negative control (i.e., “same segment but no enzyme” control) (Table 1), and the difference was not statistically significant (*P*=0.36). Also, the percentage of bound RNA11 after attempts at aminoacylation was hardly higher than even the “no RNA and no enzyme” control. Increasing the concentration of viral RNA (from 100 ng to 2.7 μg) or increasing the amount of the amino acid mixture did not have any significant effect on the readings.

**Table 1:** Binding of radiolabelled amino acids with segment 11 or HIV-2 ROD RNA in the presence or absence of HeLa S100

^14^C-labelled amino acid mix (μl)	HeLaS100 (5 mg/ml) (μl)	Yeast tRNA + HeLa S100 (% bound) (positive control)	Test RNA concentration (ng)	Segment 11 or HIV-2 ROD RNA + HeLa S100 (% bound)	Segment 11 or HIV-2 ROD^a^ RNA+ no enzyme (% bound)	No RNA + no enzyme	Fold binding of test RNA over negative control
1	2	(13.8 ± 1.8)	126 or 670^a^	(3.8 ± 2)^b^ or (2.5 ± 1.6)^a, b^	n/d	(3.0 ± 0.3)	1.3 or 0.8^a^
		(8.0 ± 0.6)	126 or 1000^a^	(1.7 ± 0.1)^b^ or (1.6 ± 0.4)^a, b^	(2.1 ± 0.7)^b^ or (1.6 ± 0.1)^a, b^	n/d	0.8 or 1.0^a^
		(9.7 ± 2.6)^b^	126	(1.4 ± 0.2)^b^	n/d	(1.7 ± 0.1)	0.8
		(13.3 ± 4) ^b^	138	(4.2 ± 1.8)^b^	n/d	2.8	1.5
				(3.0 ± 1.1)^b^	n/d	(1.5 ± 0.8)^b^	2.0
2		(13.8 ± 4.1)	500	(2.1 ± 1.1)^b^	(0.97 ± 0.2)^b^	n/d	2.2
		8.0		1.9	(0.9 ± 0.1)	n/d	2.1
		(9.5 ± 1.5)	1000	(1.3 ± 0.1)^b^	(1.2 ± 0.1)	n/d	1.1
3	3	10.3	2675	(1.4 ± 0.5)^b^	(1.7 ± 0.02)	n/d	0.8
		(8.7 ± 4.6)^b^	2675	(0.8 ± 0.6)^b^	n/d	(0.4 ± 0.05)	2.0
		(9.5 ± 4.5)^b^	2675	(1.4 ± 0.3)^b^	(1.6 ± 1.3)^b^	n/d	0.8

*n/d* not done

^**a**^Refers to data obtained using HIV-2 ROD RNA

^**b**^Mean ± standard deviation from a minimum of three replicates for each condition

Similar results were obtained using HIV-2 ROD RNA as a nonspecific control (Table 1). No relative increase in binding of RNA11 was noted compared to HIV-2 RNA when these were used side by side in the same aminoacylation experiments.

### boRV RNA11 with an added 3’ A was not a superior substrate for aminoacylation

It has been reported previously that molecules of TYMV RNAs with aminoacylable TRLS at the 3’ end could not initially be aminoacylated because 85 % of the virion RNAs terminated in 3’ CC. Once 3’-adenylated by host or other CCA-NTase (e.g., wheat germ CCA-NTase), these RNAs became competent for valylation [8].

The above aminoacylation assays were performed with original RNA11 transcripts assuming that crude HeLa S100 would initially adenylate the segments at their 3’ end (by CCA-NTase activity), followed by aminoacylation by aminoacyl synthetase activity. To facilitate the process, RNA11 was transcribed to have a 3’- CCA (see "Materials and methods") and used as a more competent substrate for the aminoacylation experiments. Several attempts using different concentrations of the 3’-adenylated RNA11 did not show increased binding to the amino acid(s) when HeLa S100 was added compared to the no-enzyme or non-adenylated RNA11 as controls (Table 2). When tested for aminoacylation, both RNA11 (Table 1, last row) and its 3’-adenylated version (Table 2, last row) produced similar readings. *In vitro* incorporation of a 5’ cap into 3’-adenylated RNA11 did not have any effect on the outcome of aminoacylation assays (data not shown).

**Table 2:** Binding of radiolabelled amino acids with segment 11-3’A (3’-adenylated) in the presence or absence of HeLa S100

^14^C-labelled amino acid mix (μl)	HeLaS100 (5 mg/ml) (μl)	Yeast tRNA + HeLa S100 (% bound) (positive control)	Test RNA concentration (ng)	Segment 11-3’A + HeLa S100 (% bound)	Segment 11-3’A or segment 11 + no enzyme (% bound)	Fold binding of test RNA over negative control
3	3	(15.4 ± 3.0)	400	2.1	3.3	<1
		10.3	2745	(3.1 ± 1.5)^a^	(1.7 ± 0.02)	1.8
		(8.7 ± 4.6)^a^	2745	(0.8 ± 0.4)^a^	(0.4 ± 0.05)	2
		(9.5 ± 4.5)^a^	2745	(1.7 ± 0.8)^a^	(1.6 ± 1.3)^a^	1

*n/d* not done

^**a**^Standard deviation from at least three replicates or independent experiments

The presence of a tRNA-like conformation has been shown to be associated with RNA packaging in plant viruses [27]. Rotavirus RNA11 has conserved sequences, particularly at the 5’ and 3’ termini [2]. A possible function of a tRNA-like conformation in RV RNA11 may be to interact with, select, and package the 10 other segments within progeny virions. Packaging signals for several segmented RNA viruses (e.g., influenza virus, bluetongue virus) have been identified in the 5’- and 3’-terminal regions, often extending into the open reading frames [28–30]. An analogous situation is described for rotavirus RNAs [2]. Thus, to avoid confounding issues of structural perturbation by central segmental regions of RV RNA11, we constructed a central deletion mutant comprising the 5’ and 3’ termini stabilised by a central UNCG loop. However, this construct did not show any evidence, either, of aminoacylation (Table 3) or 3’- adenylation (Fig. 5d) relative to the controls.

**Table 3:** Binding of radiolabelled amino acids with deletion construct of segment 11 in the presence or absence of HeLa S100

^14^C-labelled amino acid mix (μl)	HeLaS100 (5 mg/ml) (μl)	Yeast tRNA + HeLa S100 (% bound) (positive control)	Test RNA concentration (ng)	Segment 11 fragment + HeLa S100 (% bound)	Segment 11 fragment + no enzyme (% bound)	No RNA + no enzyme	Fold binding of test RNA over negative control
2	2	(13.8 ± 1.8)	90	(3.0 ± 1.7)^a^	n/d	(3.0 ± 0.3)	1
		(9.7 ± 2.6)^a^		(1.6 ± 1.0)^a^	n/d	(1.7 ± 0.1)	<1
3	3	(11.1 ± 3.4)	5000	9.1	4.7	n/d	2

*n/d* not done

^**a**^Standard deviation from at least three replicates or independent experiments

The presence of Mg^2+^ is believed to be required for optimal tRNA function, partially due to stabilisation of tertiary interactions between the loop regions [31, 32]. Interestingly, *E. coli* tRNA^Glu^ has been shown to adopt an alternative conformation (similar to a panhandle) at low Mg^2+^ concentration [33]. However, increased concentrations of Mg^2+^ in the reaction buffer did not augment *in vitro* 3’-adenylation of RNA11 (10.0-20.0 mM Mg^2+^) or aminoacylation (5.0-20.0 mM Mg^2+^) of RNA11 with 3’ A (data not shown).

### NMR spectroscopy supported a predominant panhandle structure

**Fig. 6:**
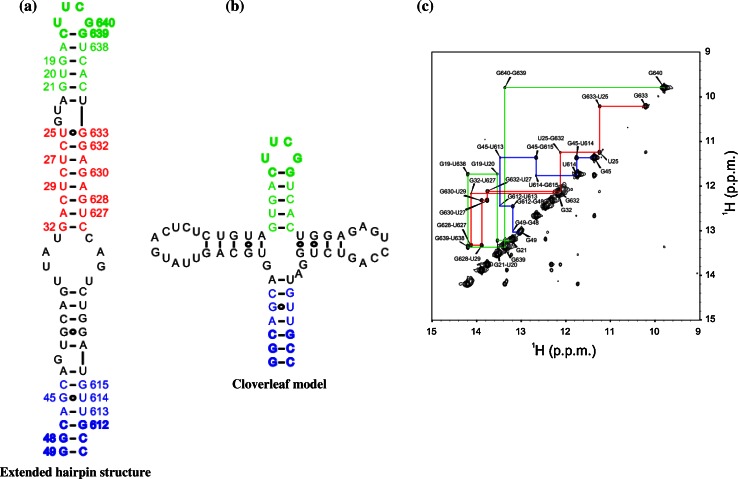
NMR-based secondary structure models of bovine rotavirus UKtc strain RNA segment 11 5’- and 3’-deletion construct joined by a UUCG loop **a** Extended hairpin model. **b** Cloverleaf model. Sequences 18-46 at the 5’ end and 613-638 at the 3’ end of RNA11 were joined by a stable tetraloop (UUCG) on one end of the helix and clamped by three G-C base pairs (shown in *bold*, *blue colour*) at the other end. **c** Contour plot of the NOESY spectra. The *coloured lines* represent cross peaks between the nucleotides identified by numbers at the point of intersections of the respective lines, and their predicted positions are shown in the hairpin model (Fig. 6a)

Chemical and enzymatic probing data did not allow us to determine the overall secondary structure of RNA11 unambiguously [2]. NMR spectroscopy was therefore used to probe the solution structure of an RNA11 fragment. NMR spectroscopy is a very powerful tool to study RNA secondary structure, since the imino proton ‘fingerprint’ region of homonuclear 2D nuclear Overhauser effect spectroscopy (NOESY) spectra between 10 and 15 ppm provides information not only about the type of base pair formed but also their sequential neighbours [18]. An RNA construct was designed comprising nt 18-46 and 613-638 of bovine rotavirus RNA11 joined by a stable tetraloop (UUCG) on one end of the helix and clamped by three G-C base pairs at the other end (Fig. 6a and b). Since large RNAs can often misfold after purification under denaturing conditions, the RNA sample was purified under native conditions using weak anion-exchange chromatography after *in vitro* transcription [17]. Size-exclusion chromatography as well as the 2D NOESY spectrum revealed a single folded RNA species in which three helical segments could be assigned unambiguously (Fig. 6c). Consecutive imino-imino NOE cross-peaks could be detected in the terminal helix (nt 44-49 and 610-615) as well as in the helix below the tetraloop (nt 635-639 and 17-21), which is compatible with both the hairpin and the cloverleaf fold (Fig. 6b). The third assigned helix comprising nt 25-32 and 626-633, on the other hand, is consistent only with the hairpin structure. Since tRNA folds can be stabilised in the presence of magnesium ions, we recorded additional 2D NOESY spectra in the presence of 10 mM MgCl_2_, but only minor chemical shift changes around G-U wobble base pairs were observed, which can bind hexahydrated magnesium ions in the major groove. Most importantly, the NOE pattern in the middle helical segment was preserved (data not shown). These data led to the conclusion that the RNA11 deletion construct under these conditions adopts an extended structure consistent with a hairpin fold.

## Discussion

Viral RNA often has to adopt a series of different structures to fulfil different functions during the viral life cycle [34]. Often, different conformers of the same molecule co-exist but may not be individually identifiable by a single probing technique [35]. To adopt different structures, viral RNA may have to be metastable and may need additional viral and cellular factors to maintain a structure of greater stability.

Previous *in silico* and *in vitro* studies supported the existence of long-range interactions between the 5’- and 3’-terminal regions of rotavirus RNA11 [2]. The new RNAalifold analysis and the additional covariation data presented here suggest the existence of a second conformation for this interaction involving a 4-helical junction, with a potential kissing-loop interaction resulting in an L-shaped three-dimensional structure resembling that of a tRNA molecule.

Certain TRLSs have been reported to be involved in viral RNA packaging; e.g., the *in vivo* packaging of BMV RNA3 is known to be dependent on a cis-acting 3’-TRLS [27]. Furthermore, TRLS in alfalfa mosaic virus (AMV) RNA have been proposed to have regulatory roles in switching between the competitive mechanisms of virus translation and replication [36]. It has been shown that the tRNA-like conformer of AMV RNAs favours virus 3’-UTR recognition by the tRNA-specific enzyme CCA-adding enzyme *in vitro* compared to the coat protein binding (CPB) conformer [37].

In the light of the above, we tested biochemically whether the predicted tRNA-like structure of RV RNA11 also has properties comparable to other viral TRLSs. However, RV RNA11 could not be aminoacylated or adenylated like tRNA or TRLSs. Despite persuasive free-energy and covariation evidence for a tRNA-like structure, the resemblance must not be adequate under the *in vitro* conditions used to make it a substrate for these modifying enzymes.

The structure mapping data and NMR data from the 5’- and 3’-deletion construct suggest that RV RNA11 terminal sequences are more stable *in vitro* as a panhandle structure rather than a cloverleaf conformation. The observed NMR data for the RNA11 construct did not conform to the NOE spectra typically observed for tRNAs [38–40]. It is important to note that NMR conditions (pH 5, low temperature) are not physiological and are designed to identify a single structure species. Thus, it is possible that the alternative cloverleaf model has a low intrinsic thermodynamic stability and can only be formed *in vivo* upon stabilization through interactions with other cellular or viral ligands or that the 5’- SL and/or the 3’- SL, but not the complete 4-helical junction, exist transiently in the folding intermediates to ensure the correct folding of RNA11. Given the strength of the phylogenetic data supported by structural predictions together with biochemical probing consistent with the existence of more than one possible terminal structure, the strong implication is that the termini of RNA11 do have alternative conformations, which may have distinct roles in the viral life cycle.
